# The Role of Glutamate Release on Voltage-Dependent Anion Channels (VDAC)-Mediated Apoptosis in an Eleven Vessel Occlusion Model in Rats

**DOI:** 10.1371/journal.pone.0015192

**Published:** 2010-12-21

**Authors:** Eunkuk Park, Gi-Ja Lee, Samjin Choi, Seok-Keun Choi, Su-Jin Chae, Sung-Wook Kang, Youngmi Kim Pak, Hun-Kuk Park

**Affiliations:** 1 Department of Biomedical Engineering, and Healthcare Industry Research Institute, College of Medicine, Kyung Hee University, Seoul, Republic of Korea; 2 Department of Medical Zoology, College of Medicine, Kyung Hee University, Seoul, Republic of Korea; 3 Department of Neurosurgery, Kyung Hee University Medical Center, Seoul, Republic of Korea; 4 Department of Physiology, College of Medicine, Kyung Hee University, Seoul, Republic of Korea; Universidade Federal do Rio de Janeiro, Brazil

## Abstract

Voltage-dependent anion channel (VDAC) is the main protein in mitochondria-mediated apoptosis, and the modulation of VDAC may be induced by the excessive release of extracellular glutamate. This study examined the role of glutamate release on VDAC-mediated apoptosis in an eleven vessel occlusion model in rats. Male Sprague-Dawley rats (250–350 g) were used for the 11 vessel occlusion ischemic model, which were induced for a 10-min transient occlusion. During the ischemic and initial reperfusion episode, the real-time monitoring of the extracellular glutamate concentration was measured using an amperometric microdialysis biosensor and the cerebral blood flow (CBF) was monitored by laser-Doppler flowmetry. To confirm neuronal apoptosis, the brains were removed 72 h after ischemia to detect the neuron-specific nuclear protein and pro-apoptotic proteins (cleaved caspase-3, VDAC, p53 and BAX). The changes in the mitochondrial morphology were measured by atomic force microscopy. A decrease in the % of CBF was observed, and an increase in glutamate release was detected after the onset of ischemia, which continued to increase during the ischemic period. A significantly higher level of glutamate release was observed in the ischemia group. The increased glutamate levels in the ischemia group resulted in the activation of VDAC and pro-apoptotic proteins in the hippocampus with morphological alterations to the mitochondria. This study suggests that an increase in glutamate release promotes VDAC-mediated apoptosis in an 11 vessel occlusion ischemic model.

## Introduction

Brain ischemia is a condition where there is insufficient cerebral blood flow (CBF), leading to a poor oxygen supply or cerebral hypoxia, which can cause the death of brain tissue, a cerebral infarction and a loss of brain function [1]. The neuronal damage by brain ischemia is affected by the extracellular concentrations of the excitatory amino acids [2]. Glutamate is the principal excitatory neurotransmitter in the brain and the excessive release of extracellular glutamate is a key factor that promotes neuronal cell death [3], [4]. Under clinical conditions, an understanding of the excitotoxic process is needed through accurate real-time measurements of the changes in the extracellular glutamate concentration during and after a range of insults that might initiate neuronal damage [5].

Measurements of neurotransmitters, particularly the release of glutamate, using microdialysis have been developed for *in vivo* analysis with continuous monitoring. Since the application of microdialysis to the research of the nervous system, it has become an important tool for monitoring brain injuries and treatments. Although measurements of the brain chemistry have become a popular method for the research into the nervous system, most studies on microdialysis focused on neuroscience and microdialysis has only recently been used in biomedical applications. Due to the successful application of real time *in vivo* monitoring of glutamate using amperometirc biosensor technology [6], a previous study reported the real-time electronic detection of the extracellular glutamate levels in a global ischemia model using either microdialysis [7] or enzyme-immobilized carbon nanotube-field effect transistor (CNT-FET) [8].

Voltage-dependent anion channel (VDAC), which is also known as mitochondrial porin, is a highly conserved large conductance anion channel that regulates the mitochondrial energy balance and the communication of the entire cell, through a common pathway for metabolite exchange between the mitochondria and cytoplasm [9], [10]. Many studies demonstrated that VDAC plays an important role in apoptosis and its contribution regulates the function of the mitochondria in cell life and death [11], [12]. During mitochondria-mediated apoptosis, morphological and biochemical alterations stimulate the release of a large number of apoptotic proteins, including tumor protein 53 (p53) [13], Bcl-2-associated X protein (Bax) [14] and Bcl-2 [15].

Recently, it was demonstrated that the extracellular glutamate release correlates with neuronal cell death in global ischemia [16]. Despite VDAC being a key player in the process of apoptotic cell death, the role of glutamate release on the function of VDAC in a global ischemia model is not completely understood. In this study, the level of glutamate release was monitored in real time and the correlation with VDAC-mediated apoptosis in an eleven vessel occlusion model in rat was evaluated.

## Results

### The levels of %CBF and glutamate release in the ischemia group

**Figure 1:**
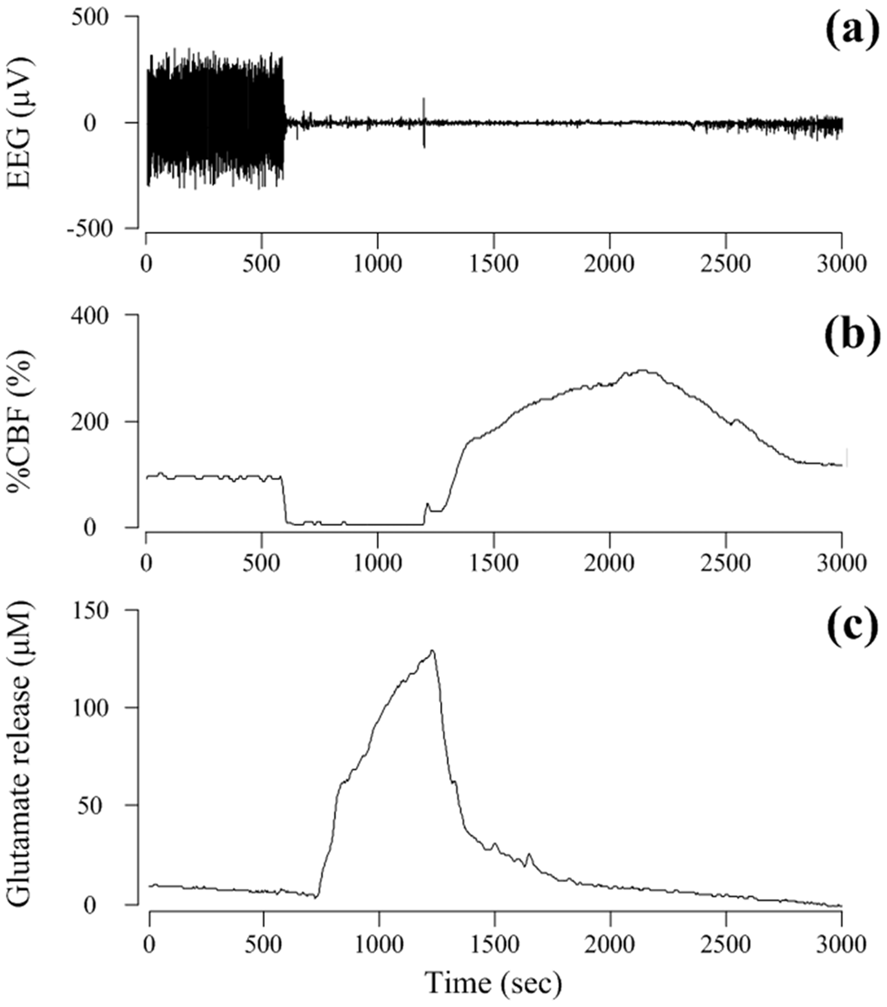
Changes in EEG, glutamate and CBF dynamics in the 11VO ischemia models.

Since the CBF is an important factor in the activation of glutamate release for cerebral brain ischemia, this study examined whether a consistently decreased CBF stimulates the release of glutamate to induce brain ischemia in an 11VO model. A similar pattern of the ischemia response was observed in the ischemia group (Fig. 1). A rapid decrease in %CBF to 12.7±2.1% of the control levels was observed after 11VO (Table 1) with the concomitant development of a flat electroencephalography (EEG) (Fig. 1). An increase in the level of glutamate release was observed from 114.9±20.2 s after the onset of ischemia with a dramatic increase occurring throughout the entire ischemic period in both groups (Table 1). In the ischemia group, the maximum change in the glutamate concentration during the ischemic period was 139.1±18.8 µM. The time elapsed from the onset of ischemia to the ischemia plateau was 14.6±3.5 s, which was maintained successfully during the ischemic period. After the initiation of reperfusion, the %CBF increased significantly to the pre-ischemic levels and the glutamate levels in the ischemia groups decreased rapidly. The time needed to reach the peak %CBF levels after the onset of the reperfusion period in the ischemia group was 691.1±246.8 s. The maximum %CBF in the ischemia group during the reperfusion period was 272.6±43.9%. After reperfusion, the peak glutamate concentration in the ischemia group was 140.7±16.7 µM.

**Table 1:** Changes in the %CBF and level of glutamate release in the ischemia group, which has been induced using an eleven vessel occlusion method.

Variable	10-minute		
Ischemic %CBF level (%)Time elapsed from the onset of ischemia to the ischemic plateau (s)	12.7±2.114.6±3.5		
Maximum level of reperfusion %CBF (%)	272.6±43.9		
Time elapsed to reach the maximum level of reperfusion %CBF after the onset of the reperfusion period (s)	691.1±246.8		
Time delay of the beginning of glutamate release after the onset of ischemia (s)		114.9±20.2	
Maximum level of ischemic glutamate release (µM)		139.1±18.8	
Maximum level of reperfusion glutamate release (µM)		140.7±16.7	

### Histological analysis

**Figure 2:**
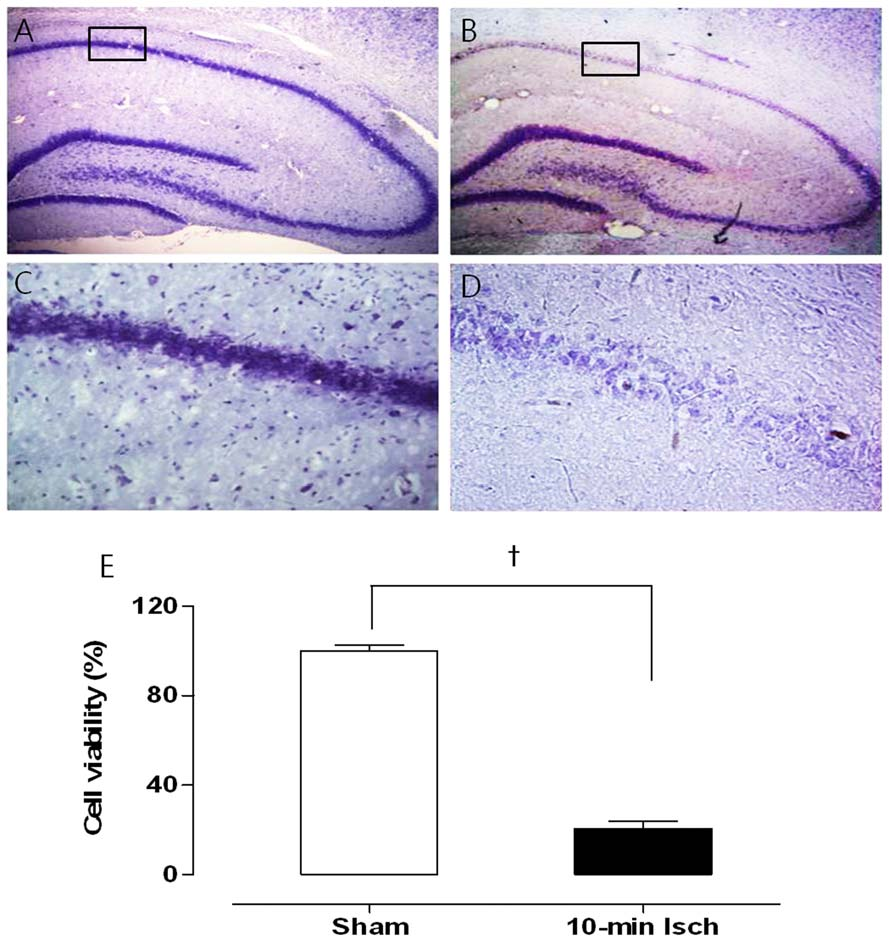
Representative morphology of Cresyl violet staining in the hippocampal region from the sham group (A and C), and ischemia group (B and D) at 72 h of reperfusion. The graph (E) shows the quantitative results of the CA1 neuronal cell counts. ^*^ *p*<0.005, ^†^ *p*<0.005.

Histological analysis of Nissl staining was performed at three days after ischemia to confirm the ischemic damage in the hippocampal region of the 11VO model. The normal pyramidal neurons from four hemispherical sections were counted and averaged. Significantly high levels of neuronal cell damage were observed in the CA1 region of the ischemia group (Fig. 2). The percentage of visible cells in the hippocampus in the ischemia group was 20.5±4.8% of that observed in the normal group.

### VDAC-mediated apoptosis

**Figure 3:**
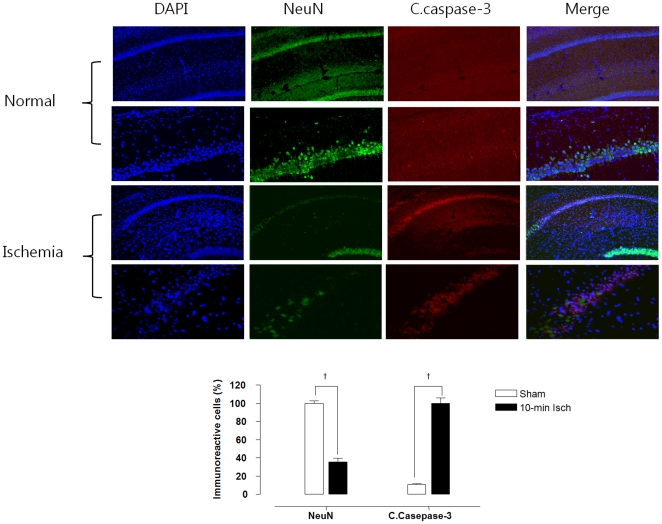
The quantitative analysis of NeuN and C.caspase-3 in the hippocampal region after global ischemia by an eleven vessel occlusion. DAPI is a fluorescent stain for both live and fixed cells. Merge between NeuN, C.caspase-3 and DIPI staining. The graph shows the quantitative results of the CA1 neuronal cell counts. ^†^ *p*<0.005.

**Figure 4:**
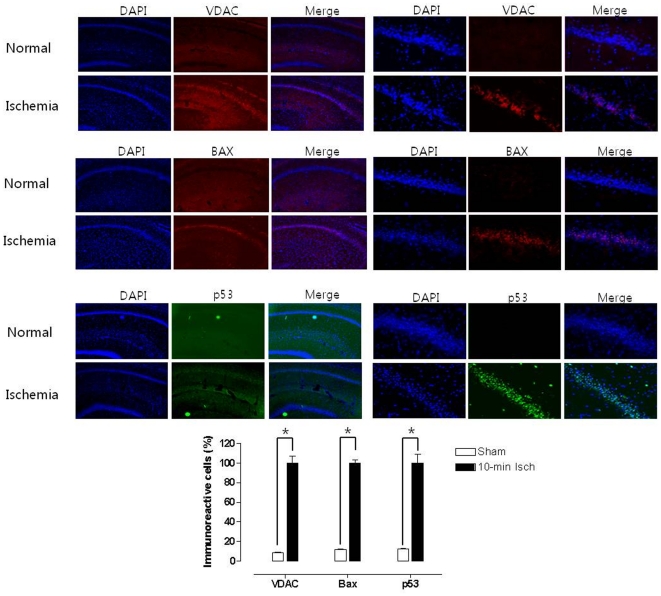
Immunoreactive cells of VDAC, BAX and p53 in the hippocampal area after eleven vessel occlusion. DAPI is a fluorescent stain both live and fixed cells. Merge between NeuN, C.caspase-3 and DIPI staining. The graph shows the quantitative results of the CA1 neuronal cell counts. ^*^ *p*<0.005,

Immune-staining for the neuronal specific nuclear protein (NeuN) and cleaved caspase-3 (C.caspase-3), VDAC, Bax and p53 in the normal and ishcemia groups were compared to confirm neuronal apoptotic cell death. The number of cells immunoreactive to NeuN was significantly higher in the normal group than in the ischemia group (36.0±4.2% of normal group; *p*<0.005). However, a larger number of cells immunoreactive to VDAC were observed in the hippocampal region of the 11VO group with the greater expression of pro-apoptotic proteins (C.caspase-3, Bax and p53) than in the normal group (Fig. 3 and 4). The immunoreactive cells from each group are presented as a percentage of the mean number of fluorescent cells. The mean number of immunoreactive cells was also obtained by three researchers blinded to the experimental conditions.

### AFM analysis

**Figure 5:**
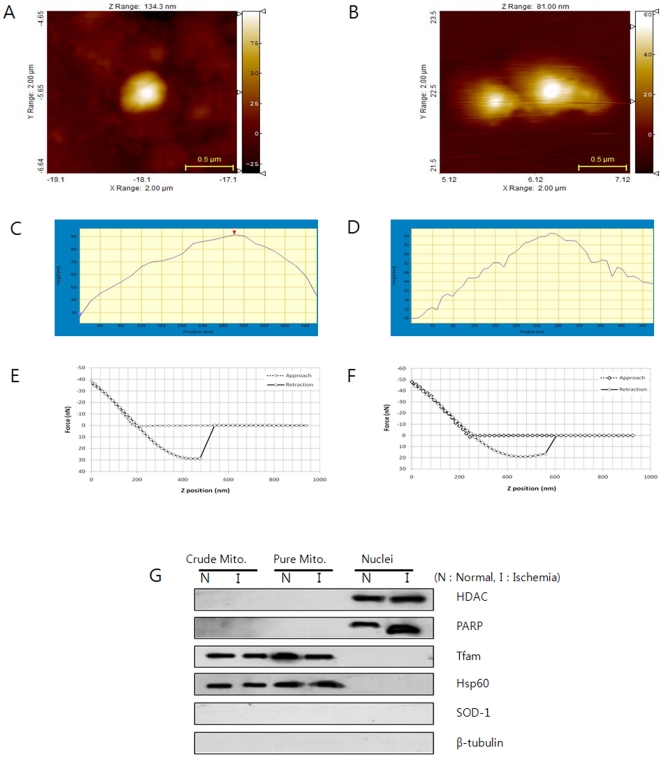
Representative AFM images (2 µm×2 µm), height profiles and force-distance curve of the mitochondria isolated from the sham group (A and C) and ischemia group (B and D) at 72 h of reperfusion. AFM mitochondrial topographical images (A and B), height profiles of AFM topographical images (C and D) and force-distance curve (E and F). Western blotting analysis (G) shows the expression of HDAC, PARP, Hsp60, Rfam, SOD-1 and β-tubulin protein in the sham and ischemia groups. Crude mito: the mitochondrial fraction isolated using the differential centrifugation method, Pure mito: mitochondrial fraction isolated using the ultracentrifugation method, Nuclei: nucleus fraction. The nucleus markers for HDAC and PARP were expressed only in the nucleus, and the mitochondria markers for Hsp60 and Tfam were detected only in the mitochondria fraction. Cytosol markers for SOD-1 and β-tubulin were not observed in the nucleus and mitochondrial fraction. Protein analysis indicates that the mitochondria are clearly isolated with no cytosol contamination.

The morphological changes to the mitochondria were observed by AFM to determine the apoptosis effect induced by ischemia-reperfusion in a global ischemia rat model. The morphology of the mitochondria after ischemia-reperfusion injury showed distinct changes compared to the normal rats (Fig. 5). The surface of the normal mitochondria was smooth and integrity. However the surface of the ischemia-reperfusion treated mitochondria became rougher than that of the normal ones. Some debris was observed around the apical end of the mitochondrial membrane. The outer membrane collapsed, and the inner membrane divided a mitochondrion into several compartments. This suggests that the debris originates from the residual cristae.

## Discussion

This is the first study to evaluate the role of glutamate in VDAC-mediated apoptosis in an eleven vessel rat occlusion model. In this study, the %CBF decreased significantly during the ischemic period, whereas an increase in glutamate release during the occlusion period was observed in the 11VO ischemia models. Three days after the ischemic occlusion, neuronal cell death was observed in the hippocampal region in the ischemia group and significant higher level of pro-apoptotic proteins along with morphological changes were observed in the ischemia group.

Brain ischemia induced by insufficient blood flow to the brain causes changes in the brain metabolism, a decrease in the metabolic rates and energy crisis [17]. Therefore, the CBF plays an important role in the development of brain ischemia. Several defects have been observed in ischemic animals, including defects in the cerebral metabolic rate with the energy state, significant death during the perfusion period [18], low survival rate for a successful ischemia study [19], and high mortality rate [20] in a 4VO model. In order to overcome these defects, the time needed to reach the ischemic plateau from the onset of ischemia with the reproducibility of ischemia plays an important role in the more close brain ischemia condition. This study demonstrated an 11VO model of rat forebrain ischemia that meets these demands [21]. Moreover, a 11VO animal model aims to design an experimental model mimicking the ischemic physiological condition with a better resolution of cerebrovascular accidents [22]. In the current study, a reproducible decrease in the %CBF after 11VO was observed with a consistent increase in glutamate release during the ischemic period. This suggests that an 11VO ischemic model may reach a level of profound reversible ischemia better mimicking complete forebrain ischemia.

A high extracellular concentration of glutamate release might play an important role in neuronal death, which is associated with a wide range of neuronal disorders [23], [24], [25]. In 1984, microdialysis was initially used to measure the level of neurotransmitters in the rat brain, suggesting that a large increase in the extracellular glutamate and aspartate content in the hippocampus is a key factor in the brain damage observed after ischemia [26]. Excitotoxic damage is affected by the glutamate release through over-activation of its receptors, such as *N*-methyl-d-aspartate (NMDA) receptors [27], and mGlu5 receptor [28]. The blockade of NMDA-receptors and mGlu5 receptor promotes a decrease in glutamate release during the ischemic period, resulting in reduced neuronal cell death in the hippocampal CA1 region. The use of microdialysis for an *in vivo* study of extracellular amino acids in the rat brain is unique method for examining the regional neurochemical events within the blood-brain barrier [29]. Next year, the first application of microdialysis in humans was carried out by detecting the interstitial glucose concentrations [30]. Currently, most microdialysis applications were used for changes in putative amino acid neurotransmitters and their metabolites in both human metabolic and animal neuroscience studies. It was reported that ischemia-induced rats during the ischemia period increases the release of extracellular glutamate, which stimulates neuronal cell death [31]. For example, glutamate concentrations >20 mM in the perfusate kills neurons in an intact brain, and the histological and ultra structural features of the glutamate lesion are similar to those of acute ischemia [32]. Microdialysis has been utilized in neuroscience to monitor the extracellular changes in the living tissue [7], and it was reported that the real time monitoring of the extracellular glutamate levels may be helpful in understanding the excitotoxic process of neurotransmitters and brain injury during or after surgery [33]. In this study, the glutamate concentration was increased steadily immediately after the occlusion during the ischemic period in the ischemia group. Three days after ischemic induction, higher levels of glutamate correlated with neuronal cell damage in the hippocampal region of the ischemia group, which concurs with a previous study [16]. Therefore, the extracellular glutamate levels, induced by 11VO might promote neuronal cell damage in the brain. In this study, an ischemic carotid artery occlusion stimulated the induction of neuronal cell death in the 11VO model.

The mitochondria are a central cellular energy source that is a crucial factor for the cellular decisions leading to apoptosis. VDAC is a major protein in mitochondria-mediated apoptosis through changes in the metabolites and energy between the cytosol and mitochondria. VDAC is believed to participate in the release of cytochrome *c* and to interact with anti- and pro-apoptotic proteins of the Bcl2 family (i.e. Bax and Bcl-xl) to their binding sites on VDAC [34]. In addition, accumulating evidence indicates that the VDAC plays a crucial role in mitochondria-mediated apoptosis. Interestingly, it was reported that VDAC possesses a specific glutamate-binding site that modulates the VDAC channel activity [35]. Despite VDAC promoting apoptosis, the relationship between VDAC and glutamate release on brain apoptosis in a global ischemia model is controversial. It was suggested that excessive glutamate levels cause neuronal apoptotic cell death, and ischemic damages in hippocampal CA1 is dependent on the release of glutamate [36]. Glutamate-induced calcium toxicity in neurons promotes changes in VDAC that are associated with mitochondrial apoptosis [37]. Furthermore, modulation of the VDAC channel activity was induced by low glutamate concentrations and the interaction of glutamate with the VDAC channel is important because VDAC is present in the plasma membrane [35].

During apoptosis, morphological and biochemical changes occur in the nucleus, cytoplasm, organelles and plasma membrane [38]. Although AFM imaging allows the supra-molecular organization of VDAC to be measured in the outer membrane of the mitochondria in yeast or potato tubes [39], [40], the mitochondrial VDAC in mammals has not been studied. The increased level of VDAC causes a rupture of the outer mitochondrial membranes, resulting in mitochondrial swelling after opening the permeability transition pores [41] as well as mitochondria-mediated apoptosis, associated with the pro-apoptotic proteins [42]. AFM imaging of the mitochondria revealed mitochondrial morphological changes by cell apoptosis. Consequently, VDAC may be essential key protein for apoptotic induction and one of the factors that alters the mitochondrial morphology.

In the present study, we present first demonstration of VDAC-mediated neuronal apoptosis with the alteration of mitochondrial morphology. Results suggest that an increase in the extracellular release of glutamate activates VDAC and pro-apoptotic proteins, leading to apoptosis. In addition, it causes morphological changes in the mitochondria. Therefore, VDAC involvement induced by excessive extracellular glutamate release in apoptotic signaling correlates with the increased expression of pro-apoptotic proteins, including p53, C.caspase-3 and BAX along with morphological changes. However, the detail apoptotic function of VDAC and its regulation by the excessive release of extracellular glutamate is unclear and requires further study.

## Materials and Methods

### Animals

Adult male Sprague-Dawley rats (Orient Bio. INC) weighing 250–350 g were kept under a 12-h light/12 h-dark cycle (lights on at 0600 h) at 24±0.5°C in a central animal care facility. Food and water were provided *ad libitum* but the animals were fasted for 1 day before the surgical procedure. All animal experiments were approved by the Committee of Animal Experiments in College of Medicine, Kyung Hee University (KHUASP(SE)-10-023), and were in strict accordance with the National Institutes of Health Guide for the Care and Use of Laboratory Animals.

During surgical preparation, the body temperature was maintained at 37.1±0.10°C using a homoeothermic blanket control unit (Harvard Apparatus, Holliston, MA, USA). The rats were anesthetized with chloral hydrate (0.1 ml/100 g, i.p.) with an additional intermittent injection (0.1 ml/300 g) for maintenance when necessary. During surgery, the arterial blood pressure was monitored using an arterial line, and the animals were not affected by the blood gas values, rectal temperature, hematocrit and hemoglobin or injury.

The rats were placed in the supine position. After the dividing the omohyoid muscle, a pair of occipital arteries, superior hypophyseal artery, ascending pharyngeal artery and pterygopalatine artery were coagulated by bipolar electrocautry. A 3 mm diameter craniotomy was drilled through the ventral clivus, centered just caual to the basioccipital suture.

The pterygopalatine arteries were coagulated before entering the tympanic bullae. Both occipital arteries and the superior thyroid arteries were identified, coagulated and transected. Snares were placed around the external carotid arteries, between the occipital arteries proximally and superior thyroid arteries distally, and the snares were placed on the common carotid arteries.

### Real-time monitoring of glutamate

The animals were placed in a stereotaxic head holder to determine the real-time glutamate levels. Changes in the CBF were monitored by laser-Doppler flowmetry with the cortical glutamate levels using a dialysis electrode. A microdialysis electrode was inserted into the motor cortex at coordinates A 1; L 4; V 2 mm (from the bregma and the dura) through a small incision in the dura. Ten-min of 11VO cerebral ischemia was initiated by pulling the snares on the CCAs and ECAs. The snares were released and withdrawn after 10-min.
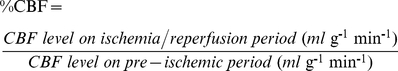



### Nissl staining

At 72 h after brain ischemia, the rat brains were fixed with cold 4% paraformaldehyde in 0.1 M phosphate buffer at pH 7.4. The brains were post-fixed overnight at 4°C in the same solution and soaked with 0.5 M phosphate buffered saline containing 30% sucrose for cryoprotection. Serial 40 µm-thick coronal sections were cut on a freezing microtome (Leica, Nussloch, Germany). The sections were stored in a cryoprotectant (25% ethylene glycol, 2 5% glycerol, 0.05 M PB, pH 7.4) at -20°C until needed.

The sections were mounted on gelatin coated slides and stained with cresyl violet for a histological assessment of neuronal cell damage, which identifies viable and nonviable stained cells. Viable neurons were defined as cells with a normal morphology, exhibiting round nuclei stained with cresyl violet. The number of viable neuronal cells in a 500 × 500 µm^2^ area of the hippocampal CA1 regions, approximately 300 µm from the hillus of the three coronal sections (approximately 1.4 to 1.8 mm posterior to bregma), was counted using Image Plus 2.0 (Motic, Xiamen, China).

### Immunofluorescence

For the detection of neuronal apoptosis, the immunofluorescence of NeuN and C.caspase-3 was performed in the hippocampal region in the sham and ischemia groups. The sections on the gelatin coated slides were incubated at room temperature for 30 min. The free-floating sections were pre-incubated for 15 min in a 1% H_2_O_2_ solution before being incubated overnight at 25°C in 0.3% Triton X-100 and 0.5 mg/ml bovine serum albumin with one of the following primary antibodies: NeuN mouse monoclonal antibody at a 1∶2000 dilution (Millipore) and caspase-3 rabbit polyclonal antibody at 1∶500 (Cell signaling), VDAC rabbit polyclonal antibody at 1∶200 (Santa Cruz), Bax rabbit polyclonal antibody at 1∶200 (Abcam) and p53 mouse monoclonal antibody at 1∶100 (Abcam). The sections were incubated with the following secondary antibodies in the dark for 60 min; Alex fluor 488 goat anti-mouse IgG antibody at 1∶1500 (Invitrogen) and Texas Red goat anti-rabbit IgG antibody at 1∶1500 (Invitrogen). After another washing period, the tissues were covered with a DAPI mounting medium (Vector).

### Preparation of mitochondria or nuclei fraction and immunoblotting

Subcellular fractions of nuclei or mitochondria were isolated by differential centrifugation, as described previously [43]. Highly-enriched mitochondria were obtained by additional ultra-centrifugation using 30–50% (1.1 and 1.6 g/ml) Optiprep™ density gradient media (Sigma-Aldrich) [43]. The purity of the mitochondria was confirmed by western blot analysis using anti-HDAC (Abcam), anti-poly (ADP-ribose) polymerase (PARP) (Santa Cruz), anti-Hsp60 (Santa Cruz), anti-mtTFA (Santa Cruz), anti-SOD1 (Santa Cruz), and anti-beta-tubulin (Abcam) antibodies, which are the markers for nuclei, mitochondria, and cytoplasm, respectively. The proteins (30 µg) were separated by 12% SDS-PAGE and transferred to a nitrocellulose membrane (Schleicher and Schuell). The membrane was incubated overnight at 4°C with the primary antibody. The HRP-conjugated secondary antibodies (Cell Signaling) followed by ECL (Amersham Biosciences Inc) were used for detection.

### AFM measurement

The mitochondrial solution was diluted with the adsorption buffer (10 mM Tris-HCl (pH 7.2), 150 mM KCl, 25 mM MgCl_2_) and dropped onto a fresh mica surface. The prepared samples were shortly air-dried at room temperature and imaged immediately by AFM. Imaging was performed in non-contact mode using a NANOS N8 NEOS microscope (Bruker, Herzogenrath, Germany) equipped with a 42.5×42.5×4 µm^3^ XYZ scanner and two Zeiss optical microscopes (Epiplan 200× and 500×). The external noise was eliminated by placing the AFM an active vibration isolation table (Table Stable Ltd., Surface Imaging Systems, Herzogenrath, Germany) inside a passive vibration isolation table (Pucotech, Seoul, Republic of Korea). The mitochondria on mica were scanned with a resolution of 512×512 pixels at a scan rate of 0.8 line/sec. Force-distance curve measurements were performed by the reflex-coated silicon cantilevers for the contact mode (PR-CO, Surface Imaging Systems, Germany) which had a spring constant of 0.2 N/m. The mitochondrial force data were obtained at locations with similar heights to avoid edge effects.

### Statistical analysis

The data for the %CBF and glutamate changes is expressed as the mean ± the standard error of the mean. A two-tailed Student's t-test was used to compare the changes in %CBF and glutamate in the two groups. The significant differences in neuronal cell viability and immunoreactive cells were assessed by one-way analysis of variance, followed by a Tukey's post hoc test using SPSS statistical software (version 17.0 for Windows, SPSS Inc., Chicage, IL). P-values <0.05 were considered significant.
